# Interleukin-27 polymorphisms are associated with premature coronary artery disease and metabolic parameters in the Mexican population: the genetics of atherosclerotic disease (GEA) Mexican study

**DOI:** 10.18632/oncotarget.16223

**Published:** 2017-03-15

**Authors:** Rosalinda Posadas-Sánchez, Nonanzit Pérez-Hernández, José Manuel Rodríguez-Pérez, Ramón M. Coral-Vázquez, Bladimir Roque-Ramírez, Luis Llorente, Guadalupe Lima, Carmina Flores-Dominguez, Teresa Villarreal-Molina, Carlos Posadas-Romero, Gilberto Vargas-Alarcón

**Affiliations:** ^1^ Departamento de Endocrinología, Instituto Nacional de Cardiología Ignacio Chávez, Mexico D.F., México; ^2^ Departamento de Biología Molecular, Instituto Nacional de Cardiología Ignacio Chávez, Mexico D.F., México; ^3^ Sección de Estudios de Posgrado e Investigación, Escuela Superior de Medicina, Instituto Politécnico Nacional, Mexico D.F., México; ^4^ Departamento de Farmacobiologia CINVESTAV-Sede Sur, Mexico D.F., México; ^5^ Departamento de Inmunología y Reumatología, Instituto Nacional de Ciencias Médicas y Nutrición Salvador Zubirán, Mexico D.F., México; ^6^ Laboratorio de Genómica Cardiovascular, Instituto Nacional de Medicina Genómica, Mexico D.F., México; ^7^ CICSA, Universidad Anahuac, Estado de México, México

**Keywords:** association studies, coronary artery disease, inflammation, interleukin 27, polymorphism genetics

## Abstract

Several studies suggest an important role of Interleukin-27 in the development of atherosclerosis. The aim of this study was to establish whether the *IL-27p28* gene polymorphisms are associated with premature coronary artery disease and/or other cardiovascular risk factors. Four *IL-27p28* gene polymorphisms were selected and genotyped in 1162 premature coronary artery disease cases and 1107 controls. rs26528 *T* and rs40837 *A* alleles were significantly associated with a lower risk of premature coronary artery disease under different inheritance models (P_dominant_ = 0.046; P_over-dominant_ = 0.002; P_co-dominant1_ = 0.007 for rs26528T; P_over-dominant_ = 0.008 and P_co-dominant1_ = 0.031 for rs40837). The rs40837 *A* allele was also associated with a lower risk of insulin resistance, in cases (P_over-dominant_ = 0.037) and controls (P_additive_ = 0.008; P_dominant_ = 0.047; P_recessive_ = 0.014; P_co-dominant2_ = 0.006), while the rs26528 *T* allele was associated with a lower risk of insulin resistance only in the control group (P_recessive_ = 0.016; P_co-dominant2_ = 0.021). Interleukin-27 plasma levels were measured in 450 controls and 450 cases, and were significantly higher in cases compared to controls (P = 0.004). However, Interleukin-27 plasma levels were not associated with *IL-27p28* polymorphisms. Luciferase assays showed that co-transfection of the rs40837 *A* allele and miR-379-5p significantly decreased luciferase gene expression. Our study shows for the first time, that IL*-27p28* gene polymorphisms are associated with premature coronary artery disease and with some metabolic parameters. The rs40837 *A* allele in presence of miR-379-5p significantly decreased luciferase gene expression.

## INTRODUCTION

Cardiovascular disease (CVD) is the main cause of morbidity in developed and emerging countries. Coronary arterial disease (CAD) is the most common CVDs mainly caused by atherosclerosis, a multifactorial disease involving both genetic and environmental factors [1]. Several studies suggest atherosclerosis could be considered an inflammatory disease [2–4]. Macrophage and T cell infiltrates are known to play an important role in atherosclerotic lesions in humans and in animal models [5,6]. Macrophages [7,8] and Th1 cells [9–11] secreted cytokines and chemokines that amplify local immune responses, while Th2 cells are known to have a protective effect on the development of atherosclerosis [12–13].

Interleukin 27 (IL-27), a member of the interleukin 12 family, is a heterodimeric cytokine, conformed by an α p28 and a β EBI3 subunit [14]. IL-27 is an immune/inflammatory response regulator [15] by promoting early Th1 differentiation [16], suppressing Th2 and Th17 differentiation [17–20], and inducing the production of anti-inflammatory cytokines such as IL-10 by activated T cell [21]. IL-27 is expressed in atherosclerotic plaques [22], and its role in atherosclerosis has been studied in cultured cells, animal models and coronary patients, with inconsistent findings. IL-27-deficient (*Ldlr*^−/−^*Ebi3*^−/−^) and IL-27 receptor-deficient (*Ldlr*^−/−^*WSX-1*^−/−^) *Ldlr*^−/−^ knockout mice were more susceptible to develop atherosclerosis, and IL-27 administration suppressed macrophage activation and atherosclerosis development [23]. However, coronary patients showed higher IL-27 levels as compared to controls, and IL-27 levels showed a significant correlation with stenosis severity [24]. Moreover, dendritic cells incubated with oxidized LDL (low density lipoprotein) produced IL-27, suggesting these modified lipoproteins could play an important role in dendritic cell activation and IL-27 production [24]. Altogether, these studies suggest IL-27 could play a crucial role in the immunity and inflammation regulatory net in atherosclerosis. The human *IL-27p28* gene encodes the IL-27 alpha subunit, located in the 16p11 locus, spans 5 exons and is highly polymorphic [25]. Considering the important role of the IL-27 in the developing of atherosclerosis, the objective of this study was to evaluate whether *IL-27p28* polymorphisms are associated with premature CAD (pCAD) and/or cardiovascular risk factors, as well as to evaluate whether the associated polymorphisms have a functional effect.

## RESULTS

### Clinical characteristics and metabolic parameters

A total of 1107 controls with no tomographic evidence [coronary artery calcification (CAC) score = 0] of subclinical atherosclerosis (SA) and 1162 pCAD cases with complete clinical, demographic, anthropometric and biochemical information belonging to the Genetics of Atherosclerotic Disease (GEA) Mexican Study were selected for the analyses. Clinical characteristics of the pCAD cases and control subjects are shown in Table 1. Age, male percentage, body mass index (BMI), waist circumference, systolic and diastolic blood pressure, visceral abdominal fat (VAF), alanine aminotransferase (ALT), aspartate aminotransferase (AST), hypoalphalipoproteinemia, hypertriglyceridemia, general obesity, abdominal obesity, type 2 diabetes mellitus (T2DM), insulin resistance, metabolic syndrome, hypertension, high VAF, hyperuricemia and hypoadiponectinemia were significantly higher in pCAD cases than in controls (Table 1). On the other hand, hypercholesterolemia [total cholesterol (TC)>200 mg/dL or low density lipoprotein-cholesterol (LDL-C) ≥130 mg/dL], high non-HDL (high density lipoprotein) cholesterol, inflammation [defined as high sensitivity C reactive protein (hsCRP) levels ≥3mg/L] and current smoking habit were significantly more frequent in controls than in pCAD cases most likely due to the effect of statin treatment (Table 1). All differences were statistically significant.

**Table 1:** Clinical characteristics and cardiovascular risk factors in the studied groups

	Controls (*n*= 1107)	pCAD (*n* = 1162)	^a^*P*
Age (years)	51 ± 9	54 ± 8	<0.001
Sex (% male)	41.2	81.1	<0.001
Body mass index (kg/m2)	27.8 [25.^4^–^30^.^8^]	28.3 [25.9-31.1]	0.003
Waist circumference (cm)	94 ± 11	98 ± 10	<0.001
Systolic blood pressure (mmHg)	102 [104-122]	116 [106-127]	<0.001
Diastolic blood pressure (mmHg)	70 [65-76]	71 [66-78]	0.013
Visceral abdominal fat (cm2)	139 [10.4-180]	168 [129-215]	<0.001
ALT (IU/L)	24 [18-34]	26 [19-36]	0.031
AST (IU/L)	25 [21-30]	26 [22-31]	0.001
Total cholesterol > 200mg/dl (%)	36.5	20.3	<0.001
LDL-C ≥ 130 mg/dL (%)	29.6	16.1	<0.001
Hypoalphalipoproteinemia (%)	52.0	67.2	<0.001
Hypertriglyceridemia (%)	47.3	56.2	<0.001
Non HDL-C>160 mg/dL (%)	27.9	19.5	<0.001
Obesity (%)	30.1	35.0	0.003
Abdominal obesity (%)	81.0	83.6	0.060
Type 2 Diabetes mellitus (%)	10.1	35.4	<0.001
Insulin resistance (%)	54.2	77.0	<0.001
Metabolic syndrome (%)	40.7	71.9	<0.001
Hypertension (%)	6.7	68.1	<0.001
Increased VAF (%)	54.5	64.6	<0.001
Current smoking habit (%)	22.8	11.6	<0.001
Hypoadiponectinemia (%)	41.5	56.5	<0.001
hsCRP ≥ 3 mg/L (%)	26.6	21.3	0.002
Hyperuricemia (%)	19.9	35.9	<0.001

Abbreviations: pCAD, premature coronary artery disease; ALT, alanine aminotransferase; AST, aspartate aminotransferase; LDL-C, low density lipoprotein cholesterol; Non HDL-C, non high density lipoprotein cholesterol; VAF, visceral abdominal fat; hsCRP, high sensitivity C reactive protein. Data are shown as average ± standard deviation, median [interquartile range] or percentage. aStudent's t test, Mann Whitney's U test or Chi square test.

### Association of IL-27 polymorphisms with pCAD

Genotype distributions of all polymorphisms in pCAD cases and controls are described in Table 2. The polymorphisms were in Hardy-Weinberg equilibrium. While genotype distributions of rs17855750 and rs181206 were similar in cases and controls, rs26528 *T* and rs40837 *A* alleles were significantly associated with a lower risk of pCAD. The rs26528 *T* allele showed significant associations with lower risk of pCAD under dominant (OR = 0.794, P = 0.046), over-dominant (OR = 0.701, P = 0.002) and co-dominant 1 (OR = 0.718, P = 0.007) models, while the rs40837 *A* allele was significantly associated with a lower risk of pCAD under the over-dominant and co-dominant 1 models (OR = 0.740, P = 0.008 and OR = 0.768, P = 0.031, respectively). All associations were adjusted for age, gender, BMI, smoking habit, total abdominal fat (TAF), homeostasis model assessment of insulin resistance (HOMA-IR), AST, adiponectin and uric acid levels (Table 2).

**Table 2:** Association of *IL-27p28* polymorphisms with premature coronary artery disease

SNP	Genotype frequency			MAF	Model	OR [95% CI]	*P*
**rs26528**	CC	CT	TT				
					Additive	0.958 [0.817-1.122]	0.594
Control (*n* = 1107)	0.378	0.481	0.142	0.382	**Dominant**	**0.794 [0.634-0.996]**	**0.046**
Patients (*n* = 1162)	0.410	0.441	0.149	0.369	Recessive	1.302 [0.958-1.771]	0.092
					**Over-dominant**	**0.701 [0.562-0.875]**	**0.002**
					**Co-dominant 1**	**0.718 [0.564-0.913]**	**0.007**
					Co-dominant 2	1.086 [0.778-1.517]	0.627
							
**rs17855750**	**AA**	**AC**	**CC**				
					Additive	0.967 [0.777-1.205]	0.767
Control (*n* = 1107)	0.729	0.248	0.023	0.147	Dominant	0.932 [0.724-1.199]	0.582
Patients (*n* = 1162)	0.731	0.242	0.024	0.146	Recessive	1.240 [0.610-2.522]	0.553
					Over-dominant	0.900 [0.694-1.169]	0.431
					Co-dominant 1	0.906 [0.697-1.177]	0.460
					Co-dominant 2	1.211 [0.594-2.470]	0.599
							
**rs181206**	**AA**	**AG**	**GG**				
					Additive	1.003 [0.855-1.178]	0.967
Control (*n* = 1107)	0.451	0.426	0.123	0.336	Dominant	0.955 [0.768-1.187]	0.677
Patients (*n* = 1162)	0.442	0.441	0.117	0.337	Recessive	1.135 [0.810-1.590]	0.463
					Over-dominant	0.923 [0.740-1.151]	0.476
					Co-dominant 1	0.936 [0.742-1.182]	0.581
					Co-dominant 2	1.073 [0.749-1.536]	0.701
							
**rs40837**	GG	GA	AA				
					Additive	0.992 [0.847-1.162]	0.923
Control (*n* = 1107)	0.378	0.480	0.143	0.382	Dominant	0.847 [0.676-1.062]	0.150
Patients (*n* = 1162)	0.405	0.445	0.150	0.372	Recessive	1.316 [0.970-1.786]	0.078
					**Over-dominant**	**0.740 [0.593-0.923]**	**0.008**
					**Co-dominant 1**	**0.768 [0.604-0.977]**	**0.031**
					Co-dominant 2	1.140 [0.818-1.589]	0.440

Abbreviations: SNP, single nucleotide polymorphism; MAF, minor allele frequency; OR, odds ratio; 95% CI, 95% confidence interval. All models were adjusted by age, gender, body mass index, smoking habit, total abdominal fat, HOMA-IR, aspartate aminotransferase, adiponectin and uric acid.

Co-dominant 1: comparison of heterozygote vs major allele homozygote genotype frequencies.

Co-dominant 2: comparison of heterozygote vs minor allele homozygote genotype frequencies.

### Associations with metabolic parameters

Tables 3 and 4 describe associations of *IL-27p28* polymorphisms with metabolic parameters in controls and pCAD cases, respectively. In the control group, rs26528 *T* and rs40837 *A* were significantly associated with a lower risk of insulin resistance (OR = 0.623, P_recessive_ = 0.016; OR = 0.610, P_co-dominant2_ = 0.021 for rs26528 *T*; OR = 0.646, P_additive_ = 0.008; OR = 0.616, P_dominant_ = 0.047; OR = 0.488, P_recessive_ =0.014 and OR = 0.404, P_co-dominant2_ = 0.006 for rs40837 *A*) and with a lower risk of increased AST activity (OR = 0.777, P_additive_ = 0.016; OR = 0.654, P_recessive_ = 0.046 and OR = 0.583, P_co-dominant2_ = 0.018 for rs26528 *T*; OR = 0.771, P_additive_ = 0.013 and OR = 0.603, P_co-dominant2_ = 0.025 for rs40837 *A*); while rs181206 *G* was associated with a lower risk of hyperuricemia (OR = 0.788, P_additive_ = 0.044) (Table 3). In pCAD cases, rs26528 *T* and rs40837 *A* were significantly associated with a lower risk of elevated AST activity (OR = 0.802, P_additive_ = 0.014; OR = 0.689, P_dominant_ = 0.003; OR = 0.737, P_over-dominant_= 0.016 and OR = 0.678, P_co-dominant1_ = 0.004 for rs26528 *T*; OR = 0.799, P_additive_ = 0.013; OR = 0.720, P_over-dominant_ = 0.009 and OR = 0.662, P_co-dominant1_ = 0.002 for rs40837 *A*). Moreover, rs181206 *G* was associated with a lower risk of hyperuricemia (OR = 0.546, P_recessive_ = 0.005 and OR = 0.566, P_co-dominant2_ = 0.011) and rs40837 *A* was associated with a lower risk of insulin resistance (OR = 0.702, P_over-dominant_ = 0.037) (Table 4).

**Table 3:** *IL27p28* gene polymorphism association with metabolic parameters in control subjects

SNP	Genotype frequency			MAF	Model	OR [95% CI]	*P*
**rs26528**	CC	CT	TT				
Insulin resistance							
No (n=507)	0.358	0.466	0.176	0.408	Recessive	0.623 [0.423-0.916]	0.016
Si (n=600)	0.395	0.492	0.113	0.359	Co-dominant 2	0.610 [0.401-0.930]	0.021
							
Aspartate aminotransferase >p75							
No (n=705)	0.368	0.471	0.161	0.396	Additive	0.777 [0.633-0.954]	0.016
Si (n=402)	0.391	0.500	0.109	0.359	Recessive	0.654 [0.431-0.992]	0.046
					Co-dominant 2	0.583 [0.373-0.912]	0.018
							
**rs181206**	AA	AG	GG				
Hyperuricemia							
No (*n* = 887)	0.438	0.434	0.128	0.345	Additive	0.788 [0.624-0.994]	0.044
Si (*n* = 220)	0.495	0.405	0.100	0.302			
							
**rs40837**	GG	GA	AA				
Insulin resistance							
No (*n* = 506)	0.354	0.470	0.176	0.411	Additive	0.646 [0.468-0.892]	0.008
Si (*n* = 601)	0.399	0.486	0.115	0.358	Dominant	0.616 [0.381-0994]	0.047
					Recessive	0.488 [0.274-0.867]	0.014
					Co-dominant 2	0.404 [0.210-0.775]	0.006
							
Aspartate aminotransferase >p75							
No (*n* = 705)	0.366	0.475	0.159	0.396	Additive	0.771 [0.628-0.947]	0.013
Si (*n* = 402)	0.339	0.489	0.112	0.357	Co-dominant 2	0.603 [0.388-0.938]	0.025

Abbreviations: SNP, single nucleotide polymorphism; MAF, minor allele frequency; OR, odds ratio; 95% CI, 95% confidence interval; p75, percentile 75. All models were adjusted by age, gender and body mass index.

Co-dominant 1: comparison of heterozygote vs major allele homozygote genotype frequencies.

Co-dominant 2: comparison of heterozygote vs minor allele homozygote genotype frequencies.

**Table 4:** *IL27p28* gene polymorphisms association with metabolic parameters in pCAD cases

SNP	Genotype frequency			MAF	Model	OR [95% CI]	*P*
**rs26528**	CC	CT	TT				
Aspartate aminotransferase>p75							
No (*n* = 710)	0.382	0.459	0.159	0.389	Additive	0.802 [0.673-0.957]	0.014
Si (*n* = 452)	0.454	0.407	0.139	0.343	Dominant	0.689 [0.539-0.882]	0.003
					Over-dominant	0.737 [0.576-0.944]	0.016
					Co-dominant 1	0.678 [0.521-0.883]	0.004
**rs181206**	AA	AG	GG				
Hyperuricemia							
No (*n* = 745)	0.439	0.426	0.135	0.348	Recessive	0.546 [0.359-0.830]	0.005
Si (*n* = 417)	0.455	0.460	0.085	0.314	Co-dominant 2	0.566 [0.365-0.878]	0.011
							
							
**rs40837**	GG	GA	AA				
Insulin resistance							
No (*n* = 316)	0.393	0.470	0.137	0.372	Over-dominant	0.702 [0.504-0.978]	0.037
Si (*n* = 846)	0.414	0.433	0.153	0.369			
							
Aspartate aminotransferase>p75							
No (*n* = 710)	0.375	0.469	0.156	0.391	Additive	0.799 [0.670-0.953]	0.013
Si (*n* = 452)	0.456	0.407	0.137	0.341	Over-dominant	0.720 [0.563-0.922]	0.009
					Co-dominant 1	0.662 [0.509-0.863]	0.002

Abbreviations: SNP, single nucleotide polymorphism; MAF, minor allele frequency; OR, odds ratio; 95% CI, 95% confidence interval; p75, percentile 75. All models were adjusted by age, gender and body mass index.

Co-dominant 1: comparison of heterozygote vs major allele homozygote genotype frequencies.

Co-dominant 2: comparison of heterozygote vs minor allele homozygote genotype frequencies.

### Haplotype analysis

Only rs40837 and rs26528 polymorphisms were in high linkage disequilibrium (r^2^>0.956). Four different haplotypes were observed, but none of them showed a significant association with pCAD (data not shown).

### IL-27 plasma levels

In a subsample, pCAD cases showed significantly higher IL-27 levels than control subjects (2.9 pg/mL vs 0.94 pg/mL, respectively; P = 0.004,). Both pCAD cases and healthy controls were non-obese individuals with hsCRP levels < 3 mg/L. IL-27 plasma levels were not significantly associated with any of the *IL-27p28* polymorphisms analyzed here.

### rs40837 *G/A* luciferase assays

**Figure 1:**
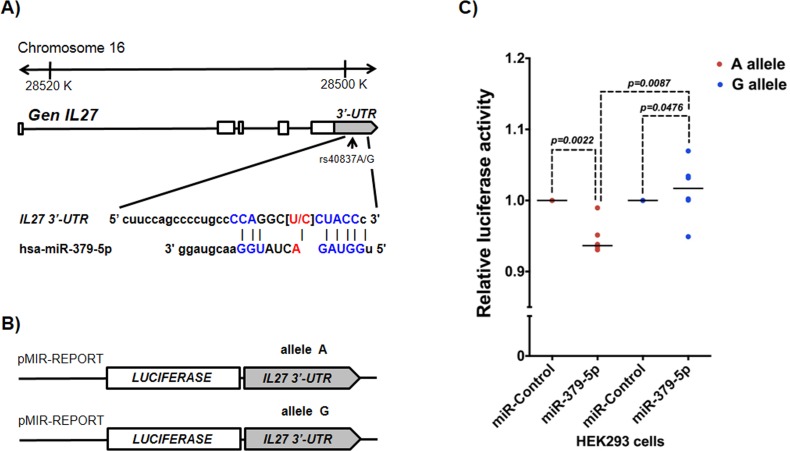
rs40837 affects luciferase expression levels in the presence of miR-379-5p **A**. Schematic diagram of the IL27 gene indicating the location of rs40837 allelic variants (*A/G*) in the 3′-UTR region and its sequence complementarity with the miR-379-5p. **B**. Sequences with each allele were cloned in pMIR-REPORT vector to evaluate the expression of the luciferase reporter gene as described in Materials and Methods. **C**. The presence of allele *A* reduced luciferase expression in the cells co-transfected with miR-379-5p as compared to cells co-transfected with miR control (*P* = 0.0022), and cells with *G* allele co-transfected with miR-379-5p (*P* = 0.0087). *n* = 5 independent duplicate experiments; medians were compared using Mann Whitney's U test.

Because rs40837 is a predicted 3′-UTR region target for miR-379-5p and/or miR-1225-5p, we examined the effect of both *G* and *A* alleles co-transfected with miR-379-5p or miR-1225-5p in a luciferase expression system. Cells co-transfected with the *A* allele construct and miR-379-5p showed a ∼5% reduction in luciferase activity (P=0.0022) compared to those co-transfected with miR-Control. In contrast, cells co-transfected with the *G* allele construct (Figure 1B) and miR-379-5p showed higher mean luciferase activity (p = 0.0476) than those cotransfected with the miR-Control (Figure 1C). Moreover, in the presence of miR-379-5p, the *A* allele construct showed significantly lower luciferase activity than the *G* allele construct (p = 0.0087). Co-transfection of both constructs (*A* or *G* allele) with the miR-1225-5p did not significantly affect luciferase expression (data not shown).

## DISCUSSION

As far as we know, this is the first study reporting the association of *IL-27p28* gene polymorphisms with pCAD. We analyzed the distribution of rs26528, rs17855750, rs181206 and rs40837 in pCAD cases and controls in order to determine whether they confer susceptibility to pCAD. Polymorphism selection was based on informatics analyses for informativity (minor allele frequency >5%) and/or predicted functional effects. Both rs26528 *T* and rs40837 *A* alleles were significantly associated with a decreased risk of developing pCAD. *IL-27p28* gene polymorphisms were also analyzed for associations with cardiometabolic risk factors, independently in cases and controls. rs26528 *T* and rs40837 *A* alleles were associated with a lower risk of high AST activity (>p75), rs181206 *G* was associated with a lower risk of hiperuricemia and rs40837 *A* with a lower risk of insulin resistance. The associations detected between the polymorphisms and cardiovascular parameters in both groups (cases and controls) were similar, suggesting that these associations are independent of the pathology present in these individuals.

Informatics analyses showed that rs40837 creates DNA binding sites for miR-379-5p and miR-1225-5p. In order to evaluate the functional effect of this polymorphism, luciferase assays were used to test the effect of each allele on gene expression in the presence of both miRNAs. In the presence of miR-379-5p, the rs40837 *A* allele showed significantly decreased luciferase gene expression. Several studies have provided evidence on the role of several miRNAs in atherosclerosis. They participate in the regulation of lipid metabolism, insulin biosynthesis, adipogenesis, endothelial dysfunction, neoangiogenesis, plaque development and rupture, as well as glucose homeostasis, among others [26-27]. Interestingly rs181206, associated with a lower risk of hyperuricemia, generates a binding site for SF/ASF proteins, which regulates alternative splicing [28]. This polymorphic site could regulate IL-27 isoforms relevant for the development of hiperuricemia.

IL-27 plays an important role in inflammation and atherosclerosis pathogenesis with dual effects, both pro and anti-inflammatory [20, 21]. It promotes early Th1 cell differentiation [16] and suppresses Th2 [18] and Th17 [19] differentiation. The type of effect on inflammation exerted by IL-27 has been reported to differ in various diseases. While it is known to promote inflammation in hepatitis [29] and systemic sclerosis [30], it suppresses inflammation in autoimmune arthritis [31], allergic asthma [32] and autoimmune encephalomyelitis [19]. Moreover, studies on the role of IL-27 in atherosclerosis in animal models and humans have shown inconsistent results. In the murine model, IL-27 administration suppressed macrophage activation and atherosclerosis development [23] and mice deficient for IL-27 or its receptor showed increased atherosclerosis susceptibility [23], suggesting IL-27 has a protective role. In contrast, coronary patients showed significantly higher IL-27 levels, which correlated with the severity of stenosis [24], suggesting a pro-atherogenic role in humans. Similar to the findings of Jin et al. [24], significantly higher IL-27 plasma concentrations were observed in Mexican pCAD cases as compared to controls.

We consider that the main strengths of this study are the following: a) The study included a large cohort of Mexican cases and controls with thorough phenotyping, and with tomographic, clinical and biochemical data, allowing to adjust our analyses for a large number of potential confounders; b) Controls included only individuals without tomographic evidence of SA (CAC score=0); c) Population stratification was ruled out as a potential confounding factor, because the proportions of Caucasian, Native American and African ancestry were similar in cases and controls; and d) A functional effect of rs40837 polymorphism on luciferase expression was observed, which was in accordance with the observed associations. Nevertheless, results should be interpreted with caution, considering the following limitations. First, due to the transversal character of the study, conclusions on causality cannot be made. Second, because the selection of participants was not random, the findings may not be applicable to the general population. However, considering that the participants have no knowledge of their genotypes, the genotype distributions would be expected to be similar in a randomly selected sample. Third, insulin resistance was not evaluated using euglycemic/hyperinsulinemic clamp, nonetheless HOMA-IR index has proven to be a reliable measurement of insulin sensitivity [33].

This study shows for the first time, that *IL-27p28* gene polymorphisms are associated with pCAD, AST activity, hyperuricemia and insulin resistance in the Mexican population. Despite all the evidence on the role of IL-27 in atherosclerosis, to best of our knowledge, to date only one cross sectional analysis has evaluated the role of *IL-27p28* gene variants in cardiovascular disease. In this study, four *IL-27p28* tag SNPs, (rs181206, rs17855750, rs37833 and rs153109) were determined in a large number of CAD cases belonging to the GeneID Chinese Han population [34]. After adjusting for confounder's variables, the polymorphisms were not associated with CAD, age at disease onset or severity [34]. These results are consistent with the findings of the present study for the rs181206 and rs17855750 *IL-27p28* gene variants. Thus, replications of the associations here reported (rs26528, rs40837) should be sought in other cohorts to confirm these results. Our results suggest rs26528*T* and rs40837A alleles could be considered as potential susceptibility markers for pCAD and insulin resistance in our population. Although these polymorphisms were not significantly associated with IL-27 plasma levels, pCAD cases showed significantly higher IL-27 levels than control subjects as previously reported [24]. The fact that the IL-27 plasma levels were not significantly associated with any of the IL-27p28 polymorphisms analyzed here could be explained considering that like other molecules, the production of IL-27 include a complex mechanism that involve not only changes at DNA level but also epigenetic modifications. Moreover, is important to consider that in our study the levels of IL-27 were measured only in a subsample of pCAD cases and controls with specific characteristics.

Because the Mexican population has particular and different genetic characteristics to other ethnic groups [35–38], the *IL-27p28* polymorphism associations observed here should be sought in other populations in order to establish if they are specific for the Mexican population or are shared with other ethnic groups.

## MATERIALS AND METHODS

### Subjects

The GEA Mexican Study was designed to examine the genetic bases of pCAD and the relationship between traditional and emerging risk factors of SA in an adult Mexican population. This study included 1200 pCAD cases and 1500 healthy individuals as control group aged 30 to 75 years. All participants were unrelated and of self-reported Mexican mestizo ancestry for 3 generations. pCAD was defined as history of myocardial infarction, angioplasty, revascularization surgery or coronary stenosis >50% on angiography, diagnosed before age 55 in men and before age 65 in women. Patients with acute cardiovascular events 3 months prior to the selection were excluded. Controls were apparently healthy asymptomatic individuals without personal or family history of pCAD, recruited from blood bank donors and through brochures posted in Social Services centers. Exclusion criteria for controls included congestive heart failure; liver, renal, thyroid or oncological disease. Standardized questionnaires were applied to all participants to obtain demographic information, family medical history, history of nutritional habits, physical activity, alcohol consumption and pharmacological treatment. The GEA study was approved by Bioethics Committee of the Instituto Nacional de Cardiología Ignacio Chávez (INCICH), and aligned to Helsinki's Declaration. All participants provided informed consent.

### Anthropometric and biochemical measurements

BMI was calculated as weight in kilograms divided by height in meters squared. Waist circumference was measured using a glass fiber measuring tape in the middle point of the distance between the lower side of the waist and the iliac crest. Blood pressure was measured at rest 3 times using a digital 5200 series Welch Allyn sphygmomanometer (Shaneateies Fails, N.Y., USA.) and the last two measurements were averaged. Venous blood samples were obtained after a 12-hour fast, and all biochemical measurements were performed at the Endocrinology Laboratory of the INCICH using standardized procedures as previously described [39–41].

### Computed axial tomography study

Computed tomography of the chest and abdomen were performed using a 64-channel multi-detector helical computed tomography system (Somatom Sensation, Siemens) and interpreted by experienced radiologists. Scans were read to assess and quantify the following: 1) CAC score using the Agatston method [42]; 2) TAF, subcutaneous and visceral abdominal fat areas (SAF and VAF) as described by Kvist [43]; and 3) hepatic to splenic attenuation ratio as described by Longo et al [44]. SA was defined as the presence CAC score>0. All pCAD cases and healthy controls underwent computed tomography. Of the 1500 apparently healthy controls, 393 subjects had a CAC score above cero, therefore they were not considered for the present analysis and were thus considered as individuals with SA. The final control group included 1107 individuals (CAC scores = 0).

### Definition of risk factors

Metabolic and cardiovascular risk factors were evaluated in both pCAD cases and controls and defined as previously described [39–41].

### Genetic analysis and functional prediction

Functional prediction of *IL-27p28* single nucleotide polymorphisms (SNPs) was sought using bioinformatics tools including FastSNP, SplicePort: An Interactive Splice Site Analysis Tool (http://spliceport.cbcb.umd.edu/SplicingAnalyser.html), SNPs3D (http://www.snps3d.org/), PESX: Putative Exonic Splicing enharcers/Silencers (http://cubweb.biology.columbia.edu/pesx/), and ESEfinder release 3.0 (http://rulai.cshl.edu/cgi-bin/tools/ESE3/esefinder.cgi).

Four IL*-27p28* gene polymorphisms with possible functional consequences and/or minor allele frequencies > 5% were selected for analysis: rs17855750 and rs181206 introduce binding sites for transcriptional factors SF2/ASF2; rs40837 modifies binding sites for miRNAs. rs26528, although not predicted as functional, was informative and in high linkage disequilibrium with rs40837 (r^2^=0.956) and was thus included in the study. Genomic DNA was extracted from peripheral blood using standard methods. All SNPs were genotyped using TaqMan assays on a real-time PCR Prism 7900HT Fast Real-Time PCR system (Applied Biosystems, Foster City, CA, USA) and analyzed by the allelic exclusion program. Samples previously sequenced of the different genotypes of the polymorphisms studied were included as positive controls.

In order to gain insight into the possible functional implications of the 3′UTR rs40837 polymorphism, the TargetScan (http://www.targetscan.org), Diana-MicroT3.0 (http://diana.cslab.ece.ntua.gr) and miRanda (http://www.microrna.org) software's were used, revealing that this SNP could be a target for miR-379-5p and miR-1225-5p.

### Estimation of ancestry

Because the Mexican-Mestizo population is admixed, in order to assess the possible influence of population stratification, a panel of 265 ancestry informative markers distinguishing mainly Amerindian, European and African ancestry were selected [45] and genotyped on Illumina BeadStation using the GoldenGate assay. Duplicate control samples were genotyped on each chip, which also served as internal controls for quality of clustering and reproducibility. The primary analysis of the genotyping data with the Illumina GenomeStudio software v.2011.1 was followed by visual inspection and assessment of data quality and clustering. Genotyping accuracy was also assessed by genotype clustering using the Illumina GeneTrain score, which is a measure of the clustering confidence of individual SNP alleles. Global Caucasian, Amerindian and African ancestry were determined using the ADMIXTURE software. Mean global ancestry was not significantly different between cases and controls (55.8% vs 54.0 % Amerindian ancestry, 34.3% vs 35.8% Caucasian and 9.8% vs 10.1% African mean ancestry for cases and controls respectively, P>0.05), strongly suggesting that population stratification was not a bias or confounding factor in this study.

### Measurement of IL-27 plasma levels

IL-27 plasma concentrations were measured in a carefully selected subsample of 450 pCAD cases and 450 healthy controls (without obesity and hsCRP < 3 mg/L), using a Bioplex system (Bio-Rad, Contra Costa County, State of California, USA) according to manufacturer's instructions.

### Reporter constructs, transfection, and luciferase assays

The 266 bp human *IL27* 3´ UTR containing the rs40837 *A* or *G* allele was amplified with the forward primer 5´-GCGCACGCGTCCCCCACCCTTTAGAACTTT-3´ and the reverse primer 5´-GCGCAAGCTTTGGATGAGAGTGCTTTATTGG-3´ from a homozygous human genomic DNA sample. PCR products were separated on agarose gels, purified and cloned into pMIR-REPORT plasmids (Applied Biosystems, Foster City, CA, USA) with MluI and HindIII digestion (Figure 1A and 1B). HEK293 cells were grown in Dulbecco's modify Eagle's medium (DMEM) (Invitrogen^TM^) supplemented with 10% fetal bovine serum and 1% antibiotics-antimycotics (Invitrogen^TM^) at 37°C with 5% CO_2_. A total of 100,000 cells were plated into each well of 12-well plates in Opti-MEM^®^ serum free medium (Invitrogen Life Technologies, Inc., Carlsbad, CA, USA). Forty-eight hours after plating, cells were co-transfected using Lipofectamine 2000 (Invitrogen, Life Technologies, Carlsbad, CA) according to the manufacturer's instructions. Each co-transfection reaction contained 500 ng of pMIR-REPORT (rs40837) *A* or *G* allele vector plus 100 ng pRL/CMV *Renilla reniformis* luciferase vector plasmids that served as a transfection internal control. Twenty-four hours after co-transfection of the plasmids, 75 nM negative control (scrambled sequence) or miR-379-5p RNA (Dharmacon GE Life Sciences) were transfected with siPORT amine transfection agent (Applied Biosystems, Foster City, CA, USA). Forty-eight hours after miRNA transfection, both *firefly* and *Renilla* luciferase activities were quantified by a dual-luciferase reporter assay system (Promega, Madison, WI). The relative luciferase activity was calculated according to the manufacturer's instructions in a TD-20/20 luminometer (Turner BioSystems, Sunnyvale, CA).

### Statistical analysis

Categorical variables were compared between groups using the Chi square test, continuous variables were compared with Student's t test or Mann Whitney's U test for parametric and non-parametric variables, respectively. Allele and genotype frequencies were estimated by direct counting. Hardy-Weinberg's equilibrium was tested using the Chi square test. For all the studied variants, statistical power to detect association with pCAD was greater than 90% as estimated with QUANTO software [http://hydra.usc.edu/GxE/]. Multivariate logistic regression analysis was used to analyze associations with pCAD under different inheritance models: additive (major allele homozygotes vs. heterozygotes vs. minor allele homozygotes), co-dominant (major allele homozygotes vs. heterozygotes and major allele homozygotes vs. minor allele homozygotes), dominant (major allele homozygotes vs. heterozygotes + minor allele homozygotes), over-dominant (heterozygotes vs. major allele homozygotes + minor allele homozygotes), and recessive (major allele homozygotes + heterozygotes vs. minor allele homozygotes). The models were adjusted for age, gender, BMI, smoking habit, TAF, HOMA-IR, AST, adiponectin and uric acid levels as appropriate. Logistic regression analyses were performed to assess associations of *IL-27* SNPs with metabolic parameters and cardiovascular risk factors, under different inheritance models and adjusting for age, gender and BMI, as appropriate. Linkage disequilibrium and haplotype analysis were performed with Haploview software (version 4.1, Broad Institute of Massachusetts Institute of Technology and Harvard University, Cambridge, MA, USA). P < 0.05 values were considered statistically significant. All analyses were performed using SPSS software v15.0 (SPSS Chicago, IL).

## Abbreviations

ALT: alanine aminotransferase
AST: aspartate aminotransferase
BMI: body mass index
CAC: coronary artery calcification
CAD: coronary artery disease
CI: confidence interval
CVD: cardiovascular disease
hsCRP: high sensitivity C reactive protein
HOMA-IR: homeostasis model assessment of insulin resistance
IL-27: interleukin 27
LDL-C: low density lipoprotein-cholesterol
MAF: minor allele frequency
OR: odds ratio
oxLDL: oxidized LDL
pCAD: premature coronary artery disease
SA: subclinical atherosclerosis
SAF: subcutaneous abdominal fat
SNP: single nucleotide polymorphism
VAF: visceral abdominal fat
TAF: total abdominal fat
TC: total cholesterol
T2DM: type 2 diabetes mellitus
